# A Pilot Study of the Usefulness of a Single Olanzapine Plasma Concentration as an Indicator of Early Drug Effect in a Small Sample of First-Episode Psychosis Patients

**DOI:** 10.1097/JCP.0000000000000770

**Published:** 2017-08-09

**Authors:** Arantzazu Zabala, Mariana Bustillo, Imanol Querejeta, Marta Alonso, Oiane Mentxaka, Ana González-Pinto, Amaia Ugarte, J. Javier Meana, Miguel Gutiérrez, Rafael Segarra

**Affiliations:** From the *Department of Neurosciences, University of the Basque Country, UPV/EHU, Bizkaia; †Early Psychosis Unit, BioCruces Health Research Institute, Barakaldo, Bizkaia; ‡Centro de Investigación Biomédica en Red de Salud Mental, CIBERSAM, Madrid; §Department of Psychiatry, Donostia University Hospital; and ∥Biodonostia Health Research Institute, Gipuzkoa; ¶Department of Psychiatry, Cruces University Hospital, Barakaldo, Bizkaia; #Department of Psychiatry, Araba University Hospital; and **BioAraba Health Research Institute, Araba; and ††Department of Pharmacology, University of the Basque Country, UPV/EHU, Bizkaia, Spain.

**Keywords:** drug effects, drug monitoring, first episode, olanzapine, pharmacokinetics

## Abstract

**Purpose/Background:**

Studies analyzing concentration-effect relationships in second-generation antipsychotics have reported contradictory results in chronic schizophrenia. No data are available for the early stages of the disease. The present study aims to evaluate the association between a single olanzapine plasma concentration, clinical response, and severity of adverse effects in first-episode psychosis (FEP); to test the utility of various plasma breakpoints as markers of early response to treatment; and to identify variables affecting olanzapine concentrations.

**Methods:**

Data from 23 compliant FEP patients receiving olanzapine monotherapy (5–30 mg/d) were evaluated 2 months after beginning treatment. Clinical symptoms were assessed using the Positive and Negative Syndrome Scale and the Montgomery-Åsberg Depression Rating Scale. Adverse effects were rated using the Udvalg for Kliniske Undersøgelser scale. Plasma samples were drawn at 11 (SD, 1) hours after dosing and analyzed with high-performance liquid chromatography/tandem mass spectrometry.

**Findings:**

Consistent with findings on chronic disease, dose, age, sex, weight, and cigarettes/day accounted for some of the variability in olanzapine concentrations. While no relationship was found between olanzapine concentrations and adverse effects or improvement of depressive symptoms, response of psychotic symptoms was associated with concentrations between 22.56 and 77.92 ng/mL. Plasma breakpoints did not show sufficiently high specificity, resulting in a large number of false-positive results.

**Implications:**

Although olanzapine concentrations do not seem to be reliable indicators of early drug effect in FEP, they may still prove useful for detecting noncompliance, as well as pharmacokinetically relevant comorbidities or genetic particularities in drug metabolism.

As a result of interindividual variability in pharmacokinetics, patients treated with the same dose can show varying plasma concentrations and, accordingly, experience a different therapeutic effect and specific pattern of adverse effects. Because drug plasma concentrations have shown a greater association with receptor occupancy than dose,^1^ it has been proposed that plasma concentrations might be a more accurate indicator of antipsychotic effects than the dose itself. Studies in the field of therapeutic drug monitoring (TDM) of olanzapine have included mainly samples of chronically ill patients with schizophrenia and reported that plasma concentrations seem to be affected by a series of factors, including age, sex, race, dose, smoking, and concomitant medication with cytochrome P450 1A2 (CYP1A2) and CYP3A4 inducers and inhibitors.^2–7^ Despite general agreement on the variables affecting olanzapine plasma concentrations, the role of TDM in the determination of clinical response or adverse effects is still dubious, given the very few and contradictory results reported in this line of research.

While several studies did not find any significant correlation between olanzapine plasma concentrations and clinical improvement,^6,8,9^ others did detect associations. Perry et al^10^ found a significant relationship between olanzapine plasma concentrations and response to treatment (≥20% decrease in Brief Psychiatric Rating Scale [BPRS] total score), identifying a concentration of 9.3 ng/mL (24 hours after administration) as a threshold for clinical response. By contrast, in the North American double-blind olanzapine trial, these same authors did not find any significant relationship between olanzapine plasma concentrations and changes in the severity of psychopathology.^9^ Nonetheless, an olanzapine concentration higher than 23.2 ng/mL (12 hours after administration) was a significant predictor of therapeutic response, defined as a decrease in total BPRS score of 20% or greater and either an end point score of 3 or less in the Clinical Global Impression Scale or a BPRS score of 35 or less. Based on receiver operating characteristic (ROC) curve analysis, Fellows et al^11^ also found that a similar breakpoint of 23 ng/mL in plasma concentrations (13.5 hours after administration) was a good threshold of clinical response (≥20% decrease in Positive and Negative Syndrome Scale [PANSS] scores). Nevertheless, even though the association found between the target plasma concentration of 23 ng/mL and clinical response was positive, it was also very modest. The leftward deflection of the ROC curve from the line of identity was small, identifying at best only 20% more responders than nonresponders. As a result, these authors recommended using TDM only if applied as a complement to the normal process of clinical evaluation.

Mauri et al^12^ found a positive correlation between dose and plasma concentrations of olanzapine and a significant curvilinear correlation between plasma concentrations and the percentage of clinical improvement (PANSS, BPRS, and Hamilton Rating Scale for Depression), associating clinical efficacy with a plasma concentration ranging between 20 and 50 ng/mL.

Lane et al^13^ found an association between plasma concentrations of olanzapine and response to treatment of depressive symptoms (≥50% reduction in the total score on the Montgomery-Åsberg Depression Rating Scale [MADRS]). The authors also maintained that an olanzapine plasma concentration of 36 ng/mL or greater (12–15 hours after administration) was able to predict mood response.

Very few studies have examined the adverse effects of olanzapine. Skogh et al^14^ found that patients who reported adverse effects had a median serum olanzapine concentration that was 22% higher than that of patients who did not experience adverse effects. This difference in median concentrations increased to 43% in patients receiving monotherapy. In contrast, other studies found no significant correlation between adverse effects and plasma concentrations of olanzapine.^11,12^

The lack of consistency in available results could be in part due to the considerable variability in methodology, such as the inclusion criteria, the heterogeneity of scales and criteria used to measure clinical response, the number of hours elapsed between administration of the last dose and blood sampling, and the diverse laboratory techniques utilized to quantify plasma concentrations. In addition, studies on TDM of second-generation antipsychotics (SGAs) in patients with first-episode psychosis (FEP) are lacking. Given the relevance of early detection and treatment of FEP to improve outcome and prevent deterioration, it seems important to investigate whether TDM is useful for clinical decision making in the early stages of the disease. Consequently, the objectives of the present study were as follows: (1) to explore the association between plasma concentrations of olanzapine, clinical response, and severity of adverse effects in FEP patients; (2) to test, in these early stages, the utility of the plasma breakpoints proposed in previous studies as markers of clinical response to treatment^9,11,13^; (3) to determine the optimum breakpoint as a marker of early response in a population of patients with FEP; and (4) to identify the demographic and clinical variables affecting olanzapine plasma concentrations.

## MATERIALS AND METHODS

The data presented here are from a 12-month prospective, naturalistic, multicenter study whose main objectives were to investigate insight and evaluate adherence in patients with FEP. The current study is focused on data from 2 months of follow-up. Nevertheless, when studying the variables of influence in plasma concentrations, data from month 6 were also considered to increase the robustness of the results.

### Participants and Pharmacological Treatment

Patients with FEP were consecutively invited to participate in the study after admission to the acute psychiatric ward of 1 of the 3 main university hospitals in the Basque Country, Spain (Cruces, Araba, and Donostia). The departments of psychiatry of these hospitals provide services to a population of approximately 1,250,000 inhabitants and cover up to 93% of the services offered to FEP patients in the Basque Country. The recruitment process lasted 52 months (September 2009 to December 2013), and patients who agreed to take part were followed up for 12 months (baseline and months 2, 6, and 12). All patients were between 18 and 50 years old, had recent-onset disease (recruitment within the first year after onset of the first positive symptom), and presented at least 1 positive symptom at the time of recruitment.

Exclusion criteria comprised a history of traumatic brain injury with loss of consciousness, organic central nervous system or pervasive developmental disorders, mental retardation (IQ <70), pregnancy/breastfeeding, or substance abuse (except tobacco) unless psychotic symptoms persisted 1 month after discontinuation.

After receiving a full explanation of the study, all participants provided their written informed consent before entry. The study was approved by the Clinical Research Ethics Committee of the Basque Country and carried out in accordance with the Declaration of Helsinki.

Once acute symptoms were stabilized, patients were discharged from the hospital. Treatment and follow-up visits were carried out as outpatients in the FEP unit of their referral hospital. Of the total of 92 FEP patients recruited, 79 (86%) were treated with a single antipsychotic drug, 11 (12%) received 2 antipsychotic drugs, and 2 patients (2%) were not taking antipsychotic medication when the first plasma sample was collected at month 2. In order to avoid potential bias arising from the effect of other antipsychotics on clinical response, only data from those patients treated with a single antipsychotic were included. Given that olanzapine was the most frequently prescribed antipsychotic agent in that subsample (n = 29 [37%]) we focused in analyzing only data from olanzapine-treated patients for whom plasma concentrations could be detected (compliant patients), resulting in a final sample of 23 FEP patients. Compliance was defined as the presence of olanzapine concentrations above the lower limit of quantitation.

Fifteen patients (65%) were treated concomitantly with other nonantipsychotic drugs. Specifically, 3 patients had benzodiazepines prescribed (diazepam, lorazepam, clonazepam, or clorazepate dipotassium), 4 a benzodiazepine and an antidepressant agent (a, diazepam and escitalopram; b, lorazepam and escitalopram; c, lormetazepam, clonazepam, and venlafaxine; or d, lorazepam, flunitrazepam, and citalopram), 1 a benzodiazepine (lormetazepam) and folic acid, and 1 a benzodiazepine (lorazepam) and a mood stabilizer (lithium). In addition, 2 patients were treated concomitantly with antidepressants only (a, escitalopram; b, escitalopram and venlafaxine), and the remaining 4 were treated with mood stabilizers (2 with lithium, 1 with valproic acid, and 1 with lithium and valproic acid).

### Clinical Assessments

Clinical data were gathered at admission and at follow-up visits. Diagnostic information was collected by trained psychiatrists using the Structured Clinical Interview for *Diagnostic and Statistical Manual of Mental Disorders, Fourth Edition, Text Revision* I at baseline. These diagnoses were confirmed after 6 and 12 months of follow-up. Psychotic and depressive symptoms were evaluated by means of the Spanish versions of PANSS^15^ and MADRS,^16^ respectively. Response to treatment of psychotic symptoms was defined as a decrease of at least 30% in the PANSS total score from baseline,^17–19^ and response to treatment of symptoms of depression as a decrease of at least 50% in the MADRS total score from baseline,^13,20^ based on the strong support of these criteria. Severity of adverse effects was assessed using the Udvalg for Kliniske Undersøgelser scale.^21^

### Plasma Sample Collection Protocol

Plasma samples were collected at steady state and routinely drawn in the early morning before breakfast at months 2, 6, and 12. At each visit, the time elapsed between last dose intake and blood sampling was recorded by the nursing staff. Blood samples were drawn 11 (SD, 1.01) hours after the last intake into 2-mL Vacutainer tubes containing K2-EDTA to prevent coagulation and, within 2 hours, centrifuged at 3000 revolutions/min (rpm) for 15 minutes to separate plasma. The supernatant was transferred to 2 × 2-mL glass vials and kept at −40°C until analysis. The plasma concentrations were unknown to the rating psychiatrists. This article focuses on analyzing samples from month 2.

### Assay of Plasma Concentrations

Once the samples had been thawed for 24 hours in a refrigerator, aliquots of 200 μL of plasma were spiked with 20 μL of 1 mg/L of haloperidol-D4 as internal standard. As a protein-precipitating agent, 600 μL of formic acid in acetonitrile (1%) was added to plasma. The samples were then mechanically shaken for 5 minutes and centrifuged at 10,000 rpm. The supernatants were transferred to Hybrid SPE cartridges. The eluates were dried and redissolved in a mobile phase. The extracts were centrifuged at 10,000 rpm at 4°C and analyzed using quantitative high-performance liquid chromatography with tandem mass spectrometry.

Chromatographic analysis was performed on an Agilent Technologies 1200 Series HPLC system (Wilmington, Del), as described in a previous article,^22^ with minor modifications. Calibration curves were prepared by spiking 20 μL of the internal standard (1 mg/L) and 20 μL of the appropriate standard working solution to obtain a final olanzapine concentration of 9.09 to 909 ng/mL.

Throughout the process, laboratory technicians were blind to the prescribed antipsychotic in each sample. All plasma samples were analyzed at random in order to test the analytical method and obtain reliable and quality results. Samples that were positive for olanzapine were reprocessed and measured before 24 hours. Given its fast degradation/oxidation, this practice ensured that an olanzapine signal was obtained.^23–25^ None of the metabolites resulting from the biotransformation of olanzapine were analyzed because of their lack of pharmacological activity.^26,27^

### Data Analysis

Mean and SDs are provided for continuous variables. The median was calculated for olanzapine concentrations because of its high variability. Discrete variables are expressed as frequencies and percentages.

For all of the analyses related to the clinical response of psychotic symptoms, a subsample of compliant patients who were in olanzapine monotherapy at month 2 was selected (n = 23). In the analyses of mood response, we selected a subsample of this group comprising only those who had a baseline MADRS score of at least 5 (n = 22), following the criteria used by Lane et al.^13^

We used nonparametric methods to evaluate the clinical response to treatment of psychotic and depressive symptoms. Differences between responders and nonresponders were calculated using Pearson χ^2^ and Mann-Whitney *U* tests. The association between plasma concentrations of olanzapine, percentage of clinical improvement (in psychotic and depressive symptoms), and severity of adverse effects was analyzed using Spearman rank correlation coefficient. In cases where a significant correlation was detected, a nonlinear regression model was constructed to further explore the relationship between the variables.

The target plasma concentrations of 23 ng/mL proposed as a marker of clinical response of psychotic symptoms^9,11^ and the breakpoint of 36 ng/mL for mood response^13^ were applied to our sample to test their quality based on their sensitivity and specificity in classifying responders and nonresponders. Nonparametric ROC curve analyses were conducted to determine the optimum breakpoints as markers of the response to treatment of psychotic and depressive symptoms. The breakpoints selected were those that struck a balance that maximized sensitivity and specificity.

To examine the variables affecting olanzapine plasma concentrations in FEP, we constructed a mixed-effects linear regression model of repeated measures including all of the available olanzapine samples from compliant patients in monotherapy at month 2 (n = 23) and month 6 (n = 18) to increase the robustness of the results. The covariates entered into the model were age, sex, dose, number of cigarettes smoked per day, weight, and cotinine concentrations. The variance-covariance matrix for olanzapine concentrations was estimated. Given the restricted sample size, the SE was estimated robustly.

All statistical tests were 2-tailed, and the level of significance was set at *P* < 0.05. STATA 13 (StataCorp, College Station, Tex) and SPSS 22 (IBM Corp, Armonk, NY) were used for the analyses.

## RESULTS

### Sample Characteristics

Demographic and clinical data are presented in Table 1. Diagnoses were grouped into 3 main categories: schizophrenia, other schizophrenia spectrum disorders, and bipolar disorder. Four patients of the sample (17%) had a diagnosis of paranoid schizophrenia. Thirteen patients (57%) received a diagnosis of a schizophrenia spectrum disorder, of whom 5 (38.5%) presented a brief psychotic disorder, 4 (30.8%) a psychotic disorder not otherwise specified, 3 (23%) a schizophreniform disorder, and the final one (7.7%) presented a schizoaffective disorder. The remaining 6 patients (26%) had a diagnosis of bipolar disorder. Nineteen of these diagnoses were confirmed after 1 year of follow-up, 2 after the first 6 months of follow-up, and the remaining 2 could not be verified beyond baseline because of loss to follow-up.

**TABLE 1:**
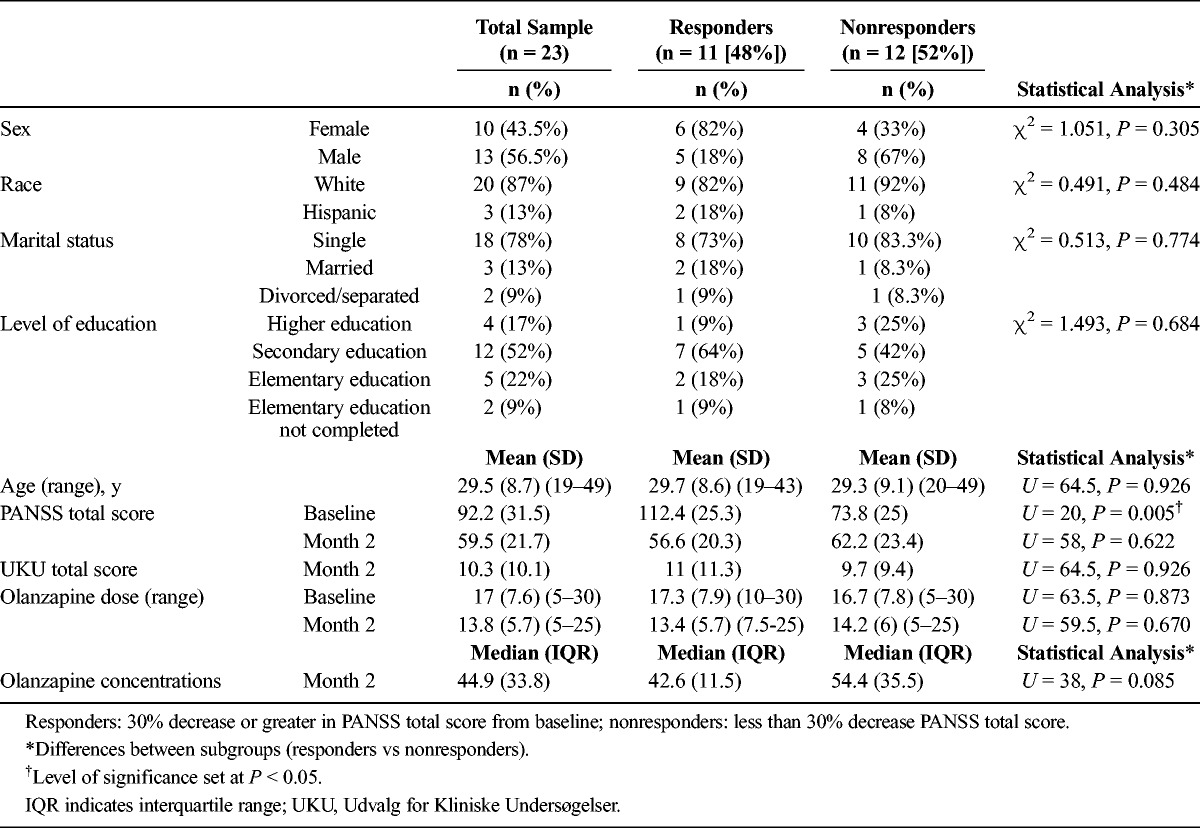
Demographic and Clinical Data of Patients in Olanzapine Monotherapy With Plasma Concentrations Detected at Month 2 (n = 23)

Fourteen patients (61%) were smokers, with a mean daily consumption of 17.2 (SD, 9) cigarettes (range, 3–40 cigarettes/day). Of these 14 patients, 6 (43%) were responders to treatment, and 8 (57%) were nonresponders. Responders smoked a mean of 15.2 (SD, 13.6) cigarettes/day (range, 3–40 cigarettes/day), whereas the mean daily consumption of nonresponders was 18.8 (SD, 3.5) cigarettes (range, 15–25 cigarettes/day). The differences in the number of cigarettes smoked per day between both groups were not significant (*U* = 13, *P* = 0.145).

When the decrease in PANSS total scores of 30% or greater was considered as the response criterion, no significant differences were detected between responders and nonresponders in demographic variables (Table 1). Likewise, there were no statistical differences between the 2 groups in dose or plasma concentrations of olanzapine. Nevertheless, responders showed higher baseline PANSS total scores than nonresponders. In fact, responders at month 2 had more severe positive symptoms upon admission (*U* = 20, *P* = 0.005), whereas nonresponders had higher scores in the general psychopathology scale of the PANSS (*U* = 20, *P* = 0.005). Still, there were no significant differences between both groups in negative symptoms (*U* = 54.5, *P* = 0.478).

When the response criterion of the decrease in MADRS scores of 50% or greater was taken into account, 8 (36%) of the 22 patients who had a baseline MADRS score of at least 5 were considered responders, and 14 (64%) were considered nonresponders. No differences were found between responders and nonresponders in demographic data, dose, or plasma concentrations. Nonetheless, a significant difference was detected between the 2 groups in the MADRS score both at baseline (*U* = 24, *P* = 0.029; responders: 29.4 [SD, 11.9], nonresponders: 19.1 [SD, 7.2]) and at month 2 (*U* = 16.5, *P* = 0.007; responders: 7.5 [SD, 5.6], nonresponders: 17.6 [SD, 9]).

#### Associations Between Plasma Concentrations, Clinical Response, and Adverse Effects

**FIGURE 1:**
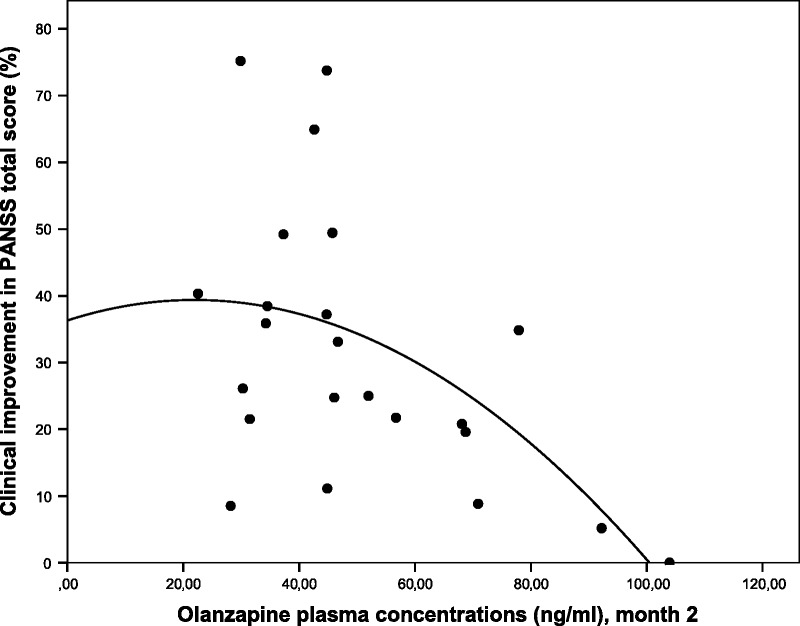
Relationship between olanzapine concentrations and improvement in PANSS total score from baseline, where *y* = 36.29 + 0.28*x* − 0.0064 × 2. Olanzapine plasma concentrations (in ng/mL), month 2.

A significant negative association between olanzapine concentrations and percentage of clinical improvement in PANSS (*r* = −0.508, *P* = 0.013) was found. Because Spearman coefficient does not assume linearity, the model with the highest *R* value was chosen after fitting different nonlinear regression models to explore this association. The model selected assumed a quadratic (curvilinear) relationship between olanzapine plasma concentrations and the percentage of improvement in PANSS total score (Fig. 1).

No association was detected between plasma concentrations and percentage of clinical improvement in MADRS scores (*r* = −0.280, *P* = 0.207) or severity of adverse effects (*r* = −0.147, *P* = 0.503).

### Identification of Responders and Nonresponders Using Plasma Breakpoints

#### Response of Psychotic Symptoms

Table 2 shows the percentages of responders and nonresponders the breakpoint of 23 ng/mL was able to classify compared with the results of Perry et al.^9^ In the present study, this breakpoint showed high sensitivity (91%) but no specificity (0%) and therefore identified 100% of false-positive results above it (ie, nonresponders).

**TABLE 2:**
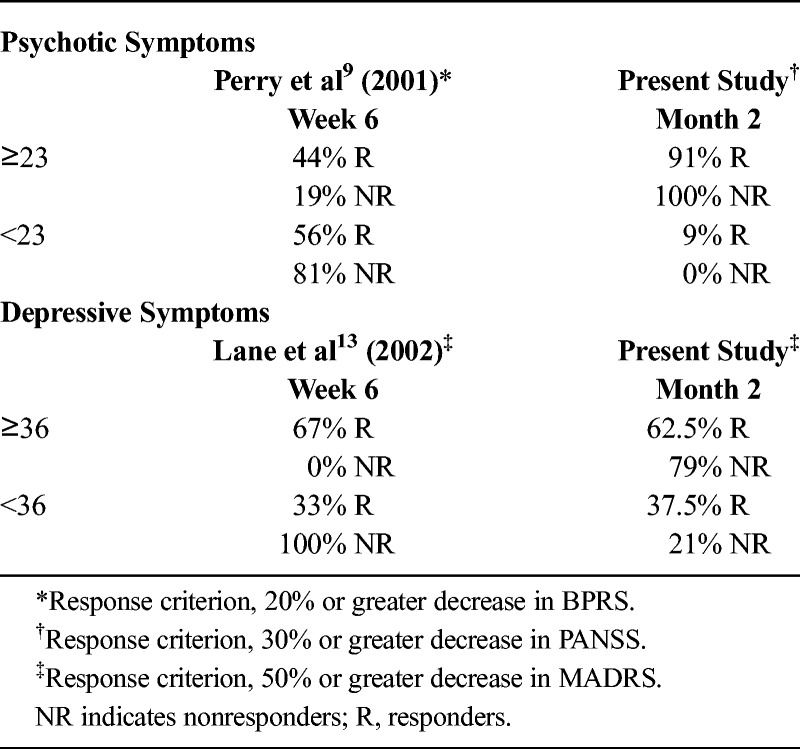
Percentage of Responders and Nonresponders Classified by the Breakpoints Proposed in the Literature for Psychotic and Depressive Symptoms

As a result, we investigated the optimum breakpoint as a marker of early response to treatment of psychotic symptoms in the study population. The breakpoint selected (34.26 ng/mL) (Table 3) showed acceptable sensitivity but poor specificity, because a higher number of false-positive results than true-negative results were detected (≥34.26 ng/mL, 75% nonresponders; <34.26 ng/mL, 25% nonresponders). Consequently, the value of the area under the curve (AUC) was too low (0.29), given that it must reach values of at least 0.6 in order to be useful.

**TABLE 3:**
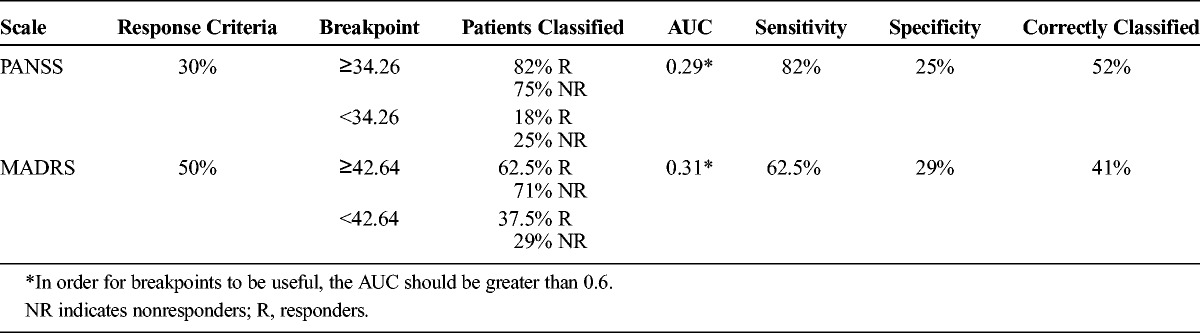
Percentage of Responders and Nonresponders Classified by the Breakpoints Obtained in the Present Study for Psychotic and Depressive Symptoms

**FIGURE 2:**
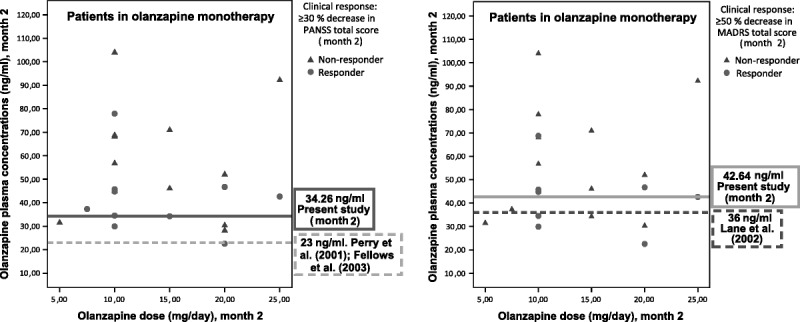
Patient distribution per dose, plasma concentrations, and clinical response of psychotic and depressive symptoms at month 2.

The breakpoints for psychotic symptoms analyzed in the present study are shown in Figure 2.

#### Response of Depressive Symptoms

Although Lane et al^13^ found the best possible specificity (100%) with a plasma breakpoint of 36 ng/mL, when this value was applied to our sample it had very low specificity (21%), detecting 79% of false-positive results above it. With respect to sensitivity, this breakpoint was able to detect a lower percentage of responders than in the original study (Table 2).

In light of this evidence, we also tried to determine the optimum breakpoint in our FEP sample. The resulting cutoff point is provided in Table 3 (42.64 ng/mL). This breakpoint had a sensitivity of 62.5%; that is, it was capable of detecting 62.5% of the responders above concentrations of 42.64 ng/mL, whereas the other 37.5% of the responders remained below this target plasma concentration (false-negative results). The specificity was 29%, leading to misclassification of nonresponders, as 71% of them had plasma concentrations of at least 42.64 ng/mL (false-positive results). Even when both breakpoints (36 and 42.64 ng/mL) were applied to our sample, the breakpoint obtained in the present study proved to be slightly better than that proposed by Lane et al,^13^ although it continued to be of little use for identifying nonresponders, provided that the percentage of nonresponders correctly classified beneath it was lower than the percentage of responders above it (false-negative results). In any case, as can be seen in Table 3, the AUC value was, once again, too low (0.31) for this breakpoint to be useful. Figure 2 shows the patient distribution per dose, plasma concentrations, and the response to treatment of depressive symptoms at month 2 with the different breakpoints analyzed in the current study, according to the 50% response criterion.

### Variables Affecting Olanzapine Pharmacokinetics in FEP

Provided that most of the patients were white (n = 20, vs Hispanic patients n = 3), and no differences were detected in plasma concentrations between the groups (*P* = 0.201), the variable race was not incorporated into the model. On the other hand, while a weak inhibition of CYP2D6 activity has been described in the literature regarding venlafaxine that could slightly increase olanzapine concentrations, patients who were treated concomitantly with this antidepressant agent (n = 2) did not differ in their olanzapine concentrations from those not taking it (*P* = 0.127). Therefore, it was not necessary to adjust the analyses for this variable. Apart from this, none of the other nonantipsychotic drugs prescribed in the sample were known to interfere with the pharmacokinetics and pharmacodynamics of olanzapine.^28,29^

The variables age, sex, weight, dose, and number of cigarettes per day proved to be significant and account in part for the variability detected in olanzapine plasma concentrations (Table 4). Older patients seem to have higher olanzapine plasma concentrations, and men tend to have lower plasma concentrations than do women. Furthermore, our data suggested that the higher the prescribed dose, the higher the plasma concentrations of olanzapine. At the same time, a higher weight was associated with higher plasma concentrations, but plasma concentrations were not related to the percentage of weight gain that occurred between baseline and month 2 (*r* = −0.2, *P* = 0.288). With respect to smoking, the greater the number of cigarettes smoked per day, the lower the concentrations of olanzapine in plasma.

**TABLE 4:**
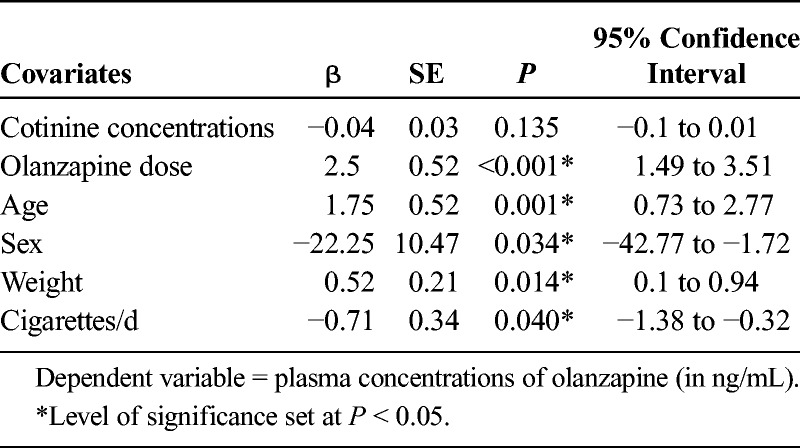
Variables Influencing Olanzapine Plasma Concentrations

## DISCUSSION

The primary finding of this study is the curvilinear relationship detected between olanzapine concentrations in FEP patients and the percentage of improvement in psychotic symptoms. No association was found between olanzapine concentrations and improvement in depressive symptoms or severity of adverse effects. As a consequence of the considerable interindividual variability observed in plasma concentrations, both the plasma breakpoints proposed by previous authors^9,11,13^ and those established in the current study showed poor specificity for discrimination between responders and nonresponders. The factors that, to some extent, explained the pharmacokinetic variability detected were age, sex, number of cigarettes smoked per day, olanzapine dose, and body weight.

The present study was based on data from the first 2 months of follow-up because of evidence suggesting that median time to response after the beginning of antipsychotic treatment in FEP patients may be as long as more than 8 weeks.^30^ Response rates in our sample at this time point were similar to those reported in other FEP studies.^31–33^ Responders had more severe symptoms upon admission and a wider range of improvement than did nonresponders, despite no differences having been detected between them for the prescribed dose. This could possibly be due to the fact that responders experienced more severe positive symptoms at baseline, on which antipsychotic treatment is more effective, but it does not have the same substantial effects on negative symptoms.^34^ In any case, results can vary considerably, depending on the response criteria chosen.^35^ This, together with the differences in the duration of follow-up periods, the pharmacological treatment administered, and the characteristics of the patients recruited, may be one of the reasons for the very different results and response rates reported in the literature.^31–33^

After testing different regression models (linear, quadratic, inverse, and logarithmic, among others) to further investigate the relationship between olanzapine concentrations and clinical improvement of psychotic symptoms, the highest *R* value was that resulting from the quadratic model (curvilinear). Mauri et al^12^ obtained a parabola similar to ours but with a lower *R* value (0.44 vs 0.53). The main difference between the parabolas of both studies is that the patients in ours experienced greater clinical improvement in PANSS total score, perhaps because they were FEP patients treated for the first time with an SGA, who are generally more sensitive to drug effect.^36^ This relationship was also curvilinear in the study by Perry et al.^10^ Nevertheless, the shape of their parabola differed from ours, and the resultant *R*^2^ value was lower than the *R*^2^ value we obtained (0.14 vs 0.281), possibly because Perry et al^10^ included an extreme value. The exclusion of that value could have generated a parabola similar to ours and to that obtained by Mauri et al.^12^

The minimum concentration that generated a response of psychotic symptoms was 22.56 ng/mL, and the maximum was 77.92 ng/mL. The Arbeitsgemeinschaft für Neuropsychopharmakologie und Pharmakopsychiatrie Therapeutic Drug Monitoring expert group, in their guidelines for TDM in psychiatry, reported a lower limit of 20 ng/mL, below which olanzapine-induced therapeutic response is relatively unlikely to occur, and an upper limit of 80 ng/mL, above which it is relatively unlikely that therapeutic improvement can be further enhanced.^37^ The curvilinear relationship found in the present study between olanzapine concentrations and clinical response is consistent with that proposal. Therefore, in FEP, clinical efficacy could be associated with an olanzapine concentration within the range of 20 to 80 ng/mL.

In contrast with the results reported by Lane et al,^13^ no association was detected between olanzapine concentrations and the percentage of improvement in depressive symptoms. This result is consistent with a recent Cochrane systematic review of the effects of SGA compared with placebo or antidepressants on major depressive disorder and dysthymia, in which olanzapine monotherapy had no beneficial effects on the treatment of depression when compared with antidepressants or placebo.^38^ Even though some FEP patients in our sample showed an improvement in psychotic symptoms, depressive symptoms may need more complex treatment. Combination of an SGA and an antidepressant could improve treatment efficacy in some cases and produce a faster relief of depressive symptoms.

Adverse effects were generally mild in our sample, and no correlation was detected with olanzapine plasma concentrations, thus supporting findings from previous studies on chronically ill patients.^11,12^ In addition to atypical antipsychotics being less frequently associated with extrapyramidal adverse effects compared with conventional neuroleptics, reasons that could explain this lack of association include the naturalistic design of the study and its non–fixed-dose regimen, which may have allowed clinicians to prescribe the most appropriate dose for each patient, thus reducing the risk of severe adverse effects.

None of the plasma breakpoints assessed were sufficiently specific to discriminate accurately between responders and nonresponders. In general, the sensitivity of all breakpoints was sufficiently high for detection of patients who responded to treatment. Nevertheless, specificity was poor, resulting in a large number of false-positive results. The breakpoint of 23 ng/mL proposed in the literature for psychotic symptoms^9,11^ proved to be too low to be useful for discriminating responders from nonresponders when applied to our sample of FEP patients. The breakpoint had high sensitivity but no specificity, and practically all the patients in the sample (responders and nonresponders) exceeded it. Even though the time interval between last intake and blood sampling was similar to that reported in the studies by Perry et al^9^ and Fellows et al,^11^ the results could have been affected by differences between those studies and the current study, namely, shorter duration of the previous studies (6 weeks maximum), use of a less strict response criterion (20% reduction in the severity of symptoms vs our 30%), and the recent onset of disease in our patients. Furthermore, in the study by Fellows et al,^11^ most of the patients were men, and this could have contributed to lower olanzapine plasma concentrations than those in our study.

Similarly, the preexisting breakpoint that Lane et al^13^ identified as a marker of mood response (36 ng/mL) was not sufficiently useful in our sample. This breakpoint had relatively poor sensitivity for identifying responders, and specificity was also too low to be able to differentiate true responders from false-positive results. Discrepancies between both studies may be accounted for by differences in ethnicity, age, and period of monitoring because the patients in the study of Lane et al^13^ were Chinese, were older (mean age, 39.1 [SD, 8.4] years), and had been followed up for only 6 weeks. In addition, the sample of the present study was somewhat larger than that of Lane et al^13^ (13 vs 22 patients with a baseline MADRS score ≥5). In addition, our study included an almost equal number of male and female patients (10 women and 12 men), whereas that of Lane et al^13^ analyzed mainly female patients (9 women and 4 men).

It is noteworthy that, in our study, nonresponders were all compliant and had higher plasma concentrations than the nonresponders from the studies of Fellows et al,^11^ Perry et al,^9^ and Lane et al.^13^ Differences in compliance between the samples could explain the high contrast in plasma concentrations because good compliance is frequently associated with higher plasma concentrations.^39^ While most nonresponders in the aforementioned studies were likely to present concentrations beneath the plasma breakpoints suggested by the authors, nonresponders in the current study tended to exhibit the highest plasma concentrations in the sample, not because of higher olanzapine doses. A possible explanation could be a higher expression of P-glycoprotein across the blood-brain barrier, which would be responsible for drug accumulation in plasma and low brain concentrations, contributing to therapeutic resistance.^40^

When determining the optimum plasma breakpoint for our own sample, the resulting breakpoints were not much better than those identified in previous studies. Plasma concentrations showed marked interindividual variability, supporting the observations of Schennach-Wolff et al^21^ regarding the problems associated with ROC analysis. Despite the frequent use of this kind of analysis, results rely too heavily on the patient sample. Consequently, small changes in plasma concentrations can drastically change the resulting breakpoint. Given that plasma concentrations change over the hours and can be affected by factors such as tobacco or caffeine,^41^ it is difficult to apply the breakpoint obtained in one study to another and even more difficult in day-to-day clinical practice. In addition, as seen in Figure 2, variations in the response criterion applied also had an effect when it came to establishing the plasma breakpoints.

As for the variables affecting plasma concentrations of olanzapine, the present results seem to be consistent with those found in previous studies on patients with chronic schizophrenia.^14,42,43^ Plasma half-life seems to be considerably longer in older patients.^4,5^ Women seem to have higher olanzapine concentrations than do men and require lower dosages to reach a specific plasma concentration.^7^ Patients with a higher weight tend to exhibit higher olanzapine concentrations, possibly because of the high liposolubility of olanzapine, whereby adipose tissue is turned into a reservoir that slowly releases the drug,^44^ leading to the prescription of higher doses. Nevertheless, the percentage of weight gain that occurred between baseline and month 2 was not related to olanzapine concentrations or to the prescribed dose. Consistent with authors who reported that smokers treated with olanzapine showed higher clearance rates than did nonsmokers after repeated oral doses,^4^ our data suggest that the higher the number of cigarettes smoked, the lower the plasma concentrations of olanzapine. This seems to be the case because agents that induce the activity of the isoenzyme CYP1A2 (eg, tobacco and carbamazepine) increase olanzapine clearance, thus reducing plasma concentrations.^14,42^ However, plasma concentrations of cotinine—nicotine's main metabolite—did not seem to have an effect on olanzapine concentrations. It has been suggested that, rather than nicotine, it is polycyclic aromatic hydrocarbons that are responsible for induction of CYP1A2 isoenzymatic activity.^45,46^

The evidence presented in this pilot study suggests that monitoring plasma concentrations does not seem to accurately quantify the effect of olanzapine on a given patient in the early stages of the illness. Despite the limited number of patients included in the sample, the present results seem to be in agreement with those of previously published studies including larger samples of chronic patients. This suggests that results are unlikely to change substantially with the inclusion of a larger number of patients. Nonetheless, to our knowledge, this is the first study to analyze the utility of olanzapine concentrations as indicators of early drug effect on FEP patients; therefore, we cannot contrast our results with previous outcomes in patients with recent-onset disease. For this reason, findings should be replicated in large-scale studies before drawing definite conclusions.

Despite the apparent poor performance of TDM for identifying the severity of adverse effects, therapeutic monitoring of SGA may still prove very useful for detecting noncompliance and thus prevent relapses and patient deterioration, given that discontinuation of antipsychotic drugs is a frequent and serious issue when treating patients with psychosis.^39,47^ In addition, TDM could provide useful information in other circumstances, such as when genetic particularities affecting drug metabolism are detected or when patients have pharmacokinetically relevant comorbidities.^37^ Because it is likely that several genes can affect the response to different drugs, future research could increase the clinical utility of TDM by including pharmacogenetic tools. Pharmacogenetic analyses performed before the initiation of treatment with SGA might help to identify which FEP patients will not respond to specific antipsychotics on the basis of their genetic profile, thus optimizing dosing and reducing the severity of adverse effects.
